# Commonalities and differences of T3SSs in rhizobia and plant pathogenic bacteria

**DOI:** 10.3389/fpls.2014.00114

**Published:** 2014-03-27

**Authors:** Anastasia P. Tampakaki

**Affiliations:** Laboratory of General and Agricultural Microbiology, Department of Crop Science, Agricultural University of AthensAthens, Greece

**Keywords:** type III secretion, plant pathogenic bacteria, rhizobia, nodulation, pili, plant-associated bacteria, translocator, atypical T3SSs

## Abstract

Plant pathogenic bacteria and rhizobia infect higher plants albeit the interactions with their hosts are principally distinct and lead to completely different phenotypic outcomes, either pathogenic or mutualistic, respectively. Bacterial protein delivery to plant host plays an essential role in determining the phenotypic outcome of plant-bacteria interactions. The involvement of type III secretion systems (T3SSs) in mediating animal- and plant-pathogen interactions was discovered in the mid-80's and is now recognized as a multiprotein nanomachine dedicated to trans-kingdom movement of effector proteins. The discovery of T3SS in bacteria with symbiotic lifestyles broadened its role beyond virulence. In most T3SS-positive bacterial pathogens, virulence is largely dependent on functional T3SSs, while in rhizobia the system is dispensable for nodulation and can affect positively or negatively the mutualistic associations with their hosts. This review focuses on recent comparative genome analyses in plant pathogens and rhizobia that uncovered similarities and variations among T3SSs in their genetic organization, regulatory networks and type III secreted proteins and discusses the evolutionary adaptations of T3SSs and type III secreted proteins that might account for the distinguishable phenotypes and host range characteristics of plant pathogens and symbionts.

## Introduction

The relationships of plants with microbes could result in different interaction outcomes, either pathogenic or mutualistic or parasitic, depending on the combination of partners. Regardless of the outcome of the association, plant-microbe interactions involve the exchange of signals between both partners. This process is highly complex and multi-layered and requires the coordination of signal sensing and regulatory networks of both partners. A common aspect of the interactions of plants with either harmful or beneficial bacteria is the recognition of microbes as invaders due to the presence of microorganism-associated molecular patterns, which results in triggering plant immune responses in order to limit microbial invasion (Boller and Felix, 2009; Zamioudis and Pieterse, 2012; Newman et al., 2013). Interestingly, microbes evolved various common mechanisms to overcome the plant immune responses and to finally establish a successful infection (Soto et al., 2006; Deakin and Broughton, 2009; Torto-Alalibo et al., 2009; Partida-Martínez and Heil, 2011; Rivas and Genin, 2011; Saeki, 2011). Despite the commonalities shared by these mechanisms, differences in the signaling molecules and networks result in the establishment either pathogenic, mutualistic or null associations.

During the last decade, numerous studies showed that microbes, including bacteria, fungi, oomycetes and nematodes deliver effector molecules either to the extracellular milieu or into the host cytoplasm to facilitate colonization. In bacteria, effector delivery occurs via diverse mechanisms such as the type III (T3SS), the type IV (T4SS), and the type VI (T6SS) secretion systems. These specialized secretion systems are thought to fine-tune the interactions of bacteria with their host partners. Of these, the T3SS has been recognized as a central player in the interaction of both pathogens and symbionts with diverse hosts. Both types of microbes exploit the T3SS to suppress or evade the plant immune system and to finally establish a successful infection.

In nowadays, the research field of type III secretion systems experiences a wave of novel insights thanks to the advances of second-generation sequencing technologies and their applications on genomics and transcriptomics. The increasing availability of whole genome sequences has revolutionized our knowledge of mechanisms that govern plant-microbe interactions. Comparative genomics and extensive genetic studies have unveiled alternative strategies used by bacteria to invade their hosts (Amadou et al., 2008; Masson-Boivin et al., 2009; Tian et al., 2012). Earlier beliefs about the specific attributes required for a particular interaction have been revised to a certain extent. For example, the absence of *nod* genes from the genomes of two photosynthetic *Bradyrhizobia* revised the assumption that *nod* genes are absolutely required for initiation of nodulation (Giraud et al., 2007). Similarly, the earlier belief that the T3SS was exclusive attribute of pathogenic bacteria was abandoned when more bacterial genome sequences became available. Moreover, the flood of sequence data has unveiled the presence of more and non-canonical T3SSs in a large number of bacteria with various lifestyles thus providing the opportunity and the challenge to gain a better understanding of the T3SS's complexity and the mechanisms underlying plant-microbe interactions and beyond.

Comparison of different genomes between phytopathogenic bacteria and rhizobia has highlighted many similarities and variations of their T3SSs. Despite the similarities among different members of plant-associated bacteria, important genetic differences also exist. This review provides a synopsis of our current understanding of the T3SSs found in rhizobia and plant pathogenic bacteria.

## General description of T3SS gene clusters in phytopathogenic bacteria and rhizobial species

**Figure 1:**
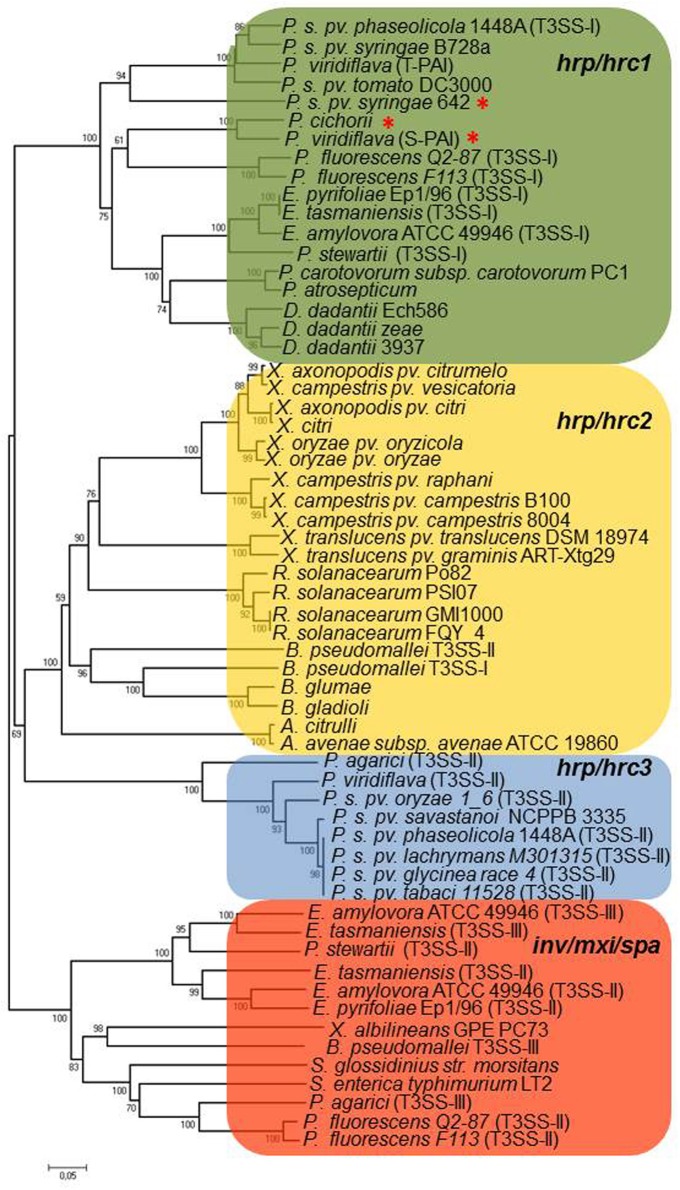
**Neighbor-joining phylogenies of SctU proteins from representative phytopathogenic and plant-associated bacteria**. The red asterisk denotes atypical *hrp*/*hrc* gene clusters, as discussed in the text. The bacteria belonging to *hrp*/*hrc3* group also harbor a *hrp*/*hrc1* gene cluster which is not represented in the tree, in all cases (see Table 1). The evolutionary distances were estimated using the p-distance method and are in the units of the number of amino acid differences per site. Numbers to the left of the branches are bootstrap values for 1000 replications. Bootstrap values greater than 50% are shown, and the scale bar represents the number of substitutions per site. Branch lengths are proportional to the amount of evolutionary change. Evolutionary analyses were conducted in MEGA6 (Tamura et al., 2013).

So far, most of the T3SS gene clusters of phytopathogenic bacteria have been divided into two groups, designated as *hrp*/*hrc1* and *hrp*/*hrc2* based on gene composition, arrangement, and transcriptional regulation but, depending on the strain, they either lack some genes or carry additional ones (Alfano and Collmer, 1997; Tampakaki et al., 2010). However, the availability of an increasing number of bacterial genomes has unveiled the presence of T3SSs with different genetic organization and phylogeny that do not fall into the abovementioned groups (Figure 1, Table 1). These novel T3SSs are discussed in detail in the following sections.The *hrp*/*hrc1* group contains clusters from species of *Pseudomonas* and *Erwinia*, while the *hrp*/*hrc2* occurs in species from *Xanthomonas*, *Ralstonia*, *Acidovorax*, and *Burkholderia*. All T3SSs are clustered within regions of ca. 22-50 kb in pathogenicity islands (PAIs), mainly located on the chromosome and in some cases on plasmids (e.g., *Ralstonia solanacearum* and *Pantoea agglomerans* pv. *gypsophilae* and pv. *betae*). The genes in both groups are organized in operons and the number of ORFs ranges from 24 (*Xanthomonas* pathovars) to 27 (*Pseudomonas* pathovars). All contain genes encoding the structural components of the secretion apparatus and secreted proteins as well as less conserved proteins with accessory roles in the structure and regulation of the secretion machinery. A unifying nomenclature has been proposed to describe the T3SS core components with the prefix secretion and cellular translocation (sct), followed by the suffixes used in the *Yersinia* Ysc system (Hueck, 1998; Abby and Rocha, 2012). Common to all functional T3SSs are proteins belonging to nine families (SctC, SctJ, SctN, SctQ, SctR, SctS, SctT, SctU, SctV). The flanking regions of T3SS clusters usually carry type III effector (T3E) genes but their gene content and structure differ among species/pathovars (Tampakaki et al., 2010). However, most effector genes are scattered throughout the genomes and are not physically linked to the T3SS gene clusters (Lindeberg et al., 2008). One common feature shared by most *Pseudomonas syringae* pathovars is the tripartite gene structure of their *hrp*/*hrc* clusters (Alfano et al., 2000). They are composed of an exchangeable effector locus (EEL), a cluster of *hrp*/*hrc* genes, and a conserved effector locus (CEL). The EEL is variable among pathovars, rich in effector-coding genes and contains many sequences related to mobile genetic elements. The CEL encodes three T3Es present in all characterized strains (Charity et al., 2003; Deng et al., 2003).

**Table 1:**
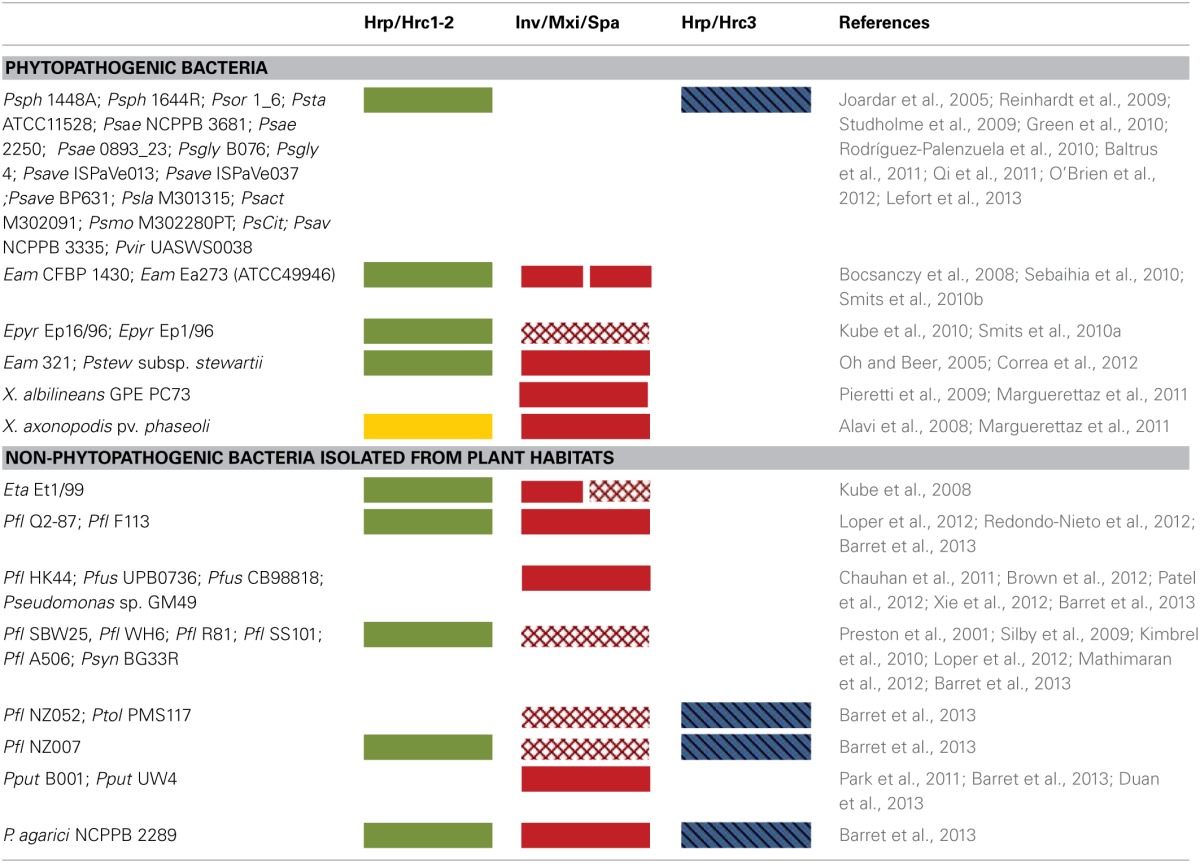
**Distribution of various T3SS types in phytopathogenic and plant-associated bacteria containing atypical T3SSs**.

Abbreviations: B. Burkholderia; E., Erwinia; Eam, E. amylovora; Epyr, E. pyrifoliae; Eta, Erwinia tasmaniensis; P., Pseudomonas; Pfl, P. fluorescens; Pfus, P. fuscovaginae; Pput, Pseudomonas putida; Ps, P. syringae; Psyn, P. synxantha; Ptol, P. tolaasii; Psph, Ps pv. phaseolicola; Psor, Ps pv. oryzae; Psta, pv. tabaci; Psae, Ps pv. aesculi; Psae, Ps pv. aesculi str. 2250E; Psgly, Ps pv. glycinea; Psave, Ps pv. avellanae; Psla, Ps pv. lachrymans; Psact, Ps pv. actinidiae; Psmo, Psmo, Ps. pv. morsprunorum; Psav, P. savastanoi pv. savastanoi; Pvir, P. viridiflava; Pstew, Pantoea stewartii subsp. stewartii; X., Xanthomonas; Colored boxes indicate the presence of a T3SS gene cluster within a genome. Each T3SS type is represented by different colors. Boxes with solid filled background indicate complete gene clusters, while boxes with light grid pattern indicate incomplete clusters. Green, yellow, red and blue boxes represent the presence of hrp/hrc1, hrp/hrc2, inv/mxi/spa and hrp/hrc3 gene clusters, respectively. The diagonal lines in the hrp/hrc3 group indicate its distinct lineage in the Rhc-II group. Two boxes in a column indicate the presence of two T3SS gene clusters.

The first evidence for the presence and functionality of T3SS in rhizobia originated from the sequencing of plasmid pNGR234a of *Sinorhizobium fredii* NGR234 (Viprey et al., 1998). Sequencing of genomes and plasmids in other rhizobial strains later revealed that T3SSs are distributed in several, but not all, rhizobial species (Kaneko et al., 2000, 2002; Göttfert et al., 2001; Krause et al., 2002; Krishnan et al., 2003; de Lyra Mdo et al., 2006). Similar to phytopathogenic bacteria, genes for T3SSs found so far in rhizobia are clustered within regions of 30–47 kb in symbiotic islands located either on the chromosome or on symbiotic plasmids (pSym), on which nodulation and nitrogen fixation genes also are located (Freiberg et al., 1997; Viprey et al., 1998; Kaneko et al., 2000; Göttfert et al., 2001; Marie et al., 2001; Krishnan et al., 2003; de Lyra Mdo et al., 2006). The genes encoding the core components of rhizobial T3SS are called *rhc* (Rhizobium conserved), while the secreted proteins are designated Nops (nodulation outer proteins) (Viprey et al., 1998; Marie et al., 2001). The rhizobial T3SS gene clusters are designated *tts* (type three secretion) and constitute a distinct family, called Rhc family (Rhizobiales family) based on phylogenetic analyses of T3S core components (Troisfontaines and Cornelis, 2005). Most of the rhizobial T3SS genes are expressed in response to plant-made flavonoids which also induce genes relevant to symbiosis (e.g., nodulation genes). Their promoter regions harbor a regulatory motif known as *tts* box, a binding site for the transcriptional activator TtsI whose production is flavonoid dependent. To date, functional T3SSs have been reported in *S. fredii* NGR234 (Viprey et al., 1998), *Bradyrhizobium japonicum* USDA110 (Krause et al., 2002), *S. fredii* USDA257 (Krishnan et al., 2003), *S. fredii* HH103 (de Lyra Mdo et al., 2006), *Bradyrhizobium elkanii* (Okazaki et al., 2009), and *Mesorhizobium loti* MAFF303099 (Okazaki et al., 2010).

In contrast to biotrophic phytopathogenic bacteria, the T3SSs are not essential for rhizobia to interact with plant hosts since several rhizobia lacking a T3SS are still able to nodulate legumes. Nevertheless, functional studies in T3SS-containing rhizobia have demonstrated the contribution of T3SS in nodule formation on several legume species. Moreover, it is of great interest the discovery of *R* genes that control symbiotic specificity at genotype level which implies that common recognition principles govern both plant-pathogen and legume-rhizobium interactions (Yang et al., 2010a).

Although substantial advances have been made in the structure, function and regulation of T3SSs in phytopathogenic bacteria, even less is known about T3SSs in rhizobia. In order to delineate the similarities and differences between T3SSs of phytopathogenic bacteria and rhizobia, it was advisable to present here the various types of rhizobial T3SSs in more detail since no overview articles in the literature are currently available for the reader.

## Rhizobial T3SS gene clusters

**Figure 2:**
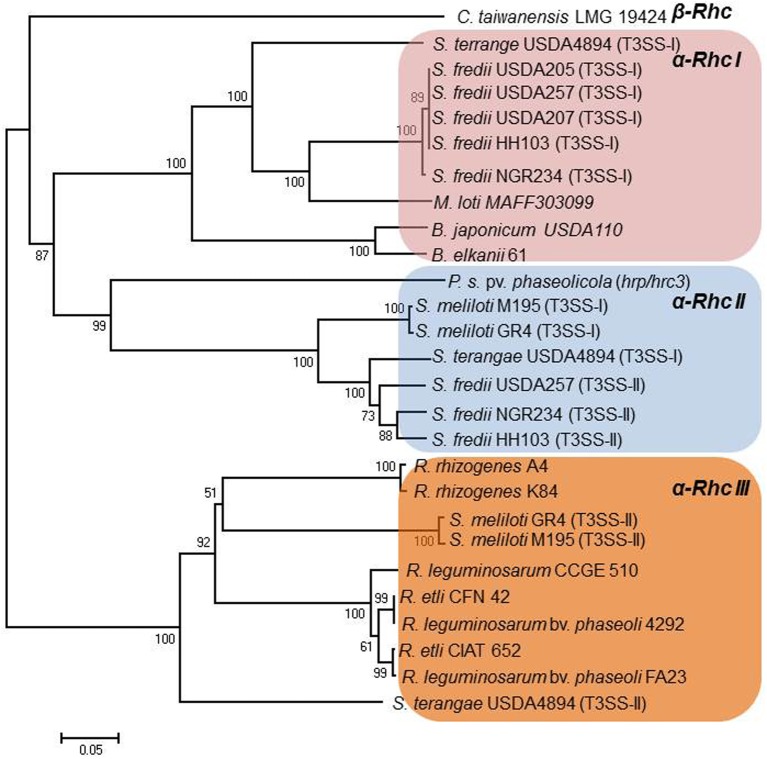
**Neighbor-joining phylogenies of SctU proteins from rhizobia**. The evolutionary distances were estimated using the p-distance method and are in the units of the number of amino acid differences per site. Numbers to the left of the branches are bootstrap values for 1000 replications. Bootstrap values greater than 50% are shown, and the scale bar represents the number of substitutions per site. Branch lengths are proportional to the amount of evolutionary change. Evolutionary analyses were conducted in MEGA6 (Tamura et al., 2013).

The Rhc T3SS family is further subdivided into four subgroups based on a phylogenetic analysis of T3SS core proteins (Gazi et al., 2012). Subgroup Rhc-I contains members of *S. fredii*, *M. loti*, and *B. japonicum*, Rhc-II involves the second T3SS of *S. fredii* NGR234 located on plasmid pNGR234b and T3SSs from various *P. syringae* pathovars, and Rhc-III is represented by the T3SSs of *R. etli* strains (Figure 2, Table 2). A distinct group designated β-Rhc forms the β-rhizobium *Cupriavidus taiwanensis*. Despite the categorization of *tts* clusters in Rhc subgroups, members belonging to the subgroup Rhc-I show subtle differences in respect to their physical gene organization and gene content. So far, only the *tts* clusters of Rhc-I group have been shown to be functional and to affect the nodulation process. Moreover, the operon structure presented here is based on the literature as was deduced from the presence of the *tts* or other motifs or the flavonoid-dependent induction of *tts*-containing promoters analyzed in many cases by transcriptional fusion reporters.

**Table 2:**
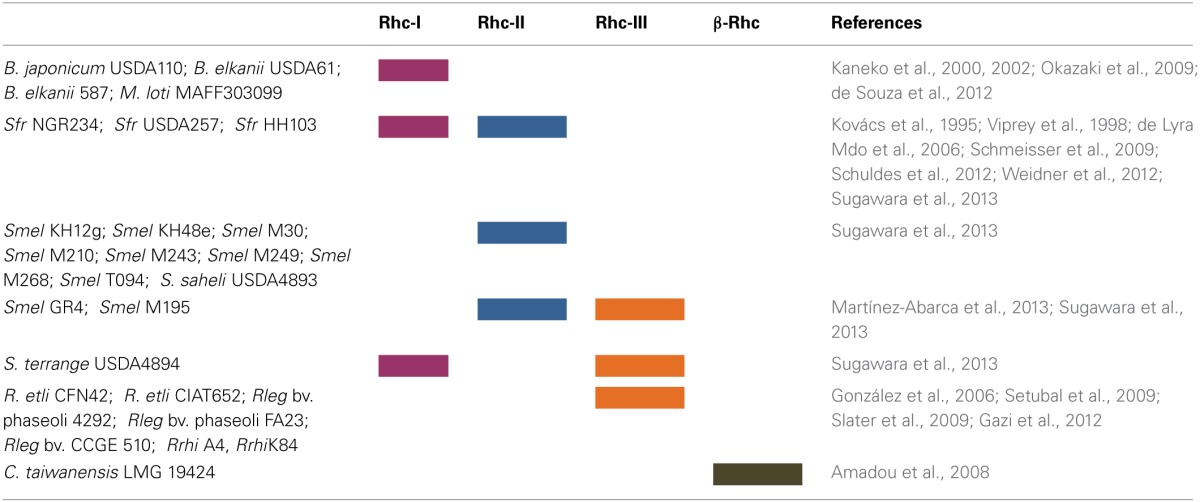
**Distribution of Rhc-T3SS subgroups in rhizobia**.

Abbreviations: S., Sinorhizobium; M., Mesorhizobium; B., Bradyrhizobium, R., Rhizobium; Sfr, S. fredii; Smel, S. meliloti; Rleg, R. leguminosarum; Rrhi, R. rhizogenes; C., Cupriavidus; Colored boxes represent the presence of a T3SS gene cluster within a genome and the different colors correspond to the various Rhc subgroups.

### Rhc-I gene clusters

**Figure 3:**
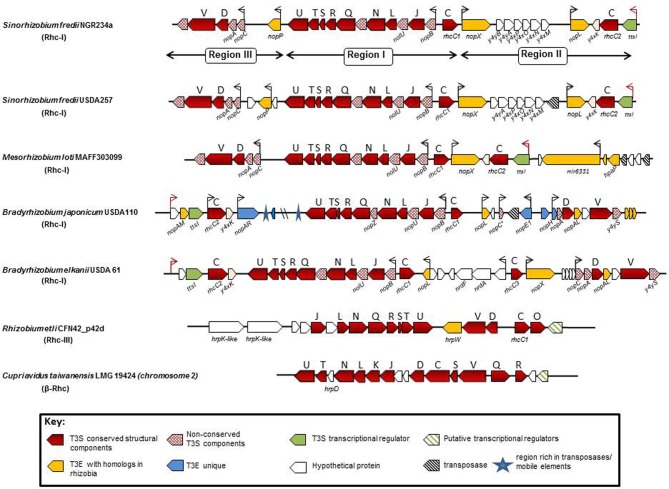
**Genetic organization of functional rhizobial T3SS gene clusters**. Genes are represented by colored arrows showing the sense of transcription. Conserved *rhc* genes are shown as red arrows and the capital letters above the arrows are abbreviations of the T3SS families according to the *sct* nomenclature. Red dotted arrows show rhizobial-specific T3SS genes. Gene names below the arrows generally correspond to nomenclature from the literature. *nod* boxes are indicated by red arrowheads and *tts* boxes by black arrowheads. Yellow arrows show type III effector genes (T3E) with homologs in other rhizobia and/or pathogenic bacteria, while blue arrows indicate T3E genes unique in the corresponding strain/species and the green ones show the genes coding for the transcriptional regulator of rhizobial T3SSs. Open arrows indicate unknown or possibly unrelated T3SS genes. Black dashed arrows show transposases/integrases and blue asterisks show regions rich in mobile elements. The locus tags of each *tts* cluster are as follows: *S. fredii* NGR234a: NGR_a00520-NGR_a00800; *S. fredii* USDA257: USDA257_p02960-USDA257_p02630; *M. loti:* mlr6327-mlr8765; *B. japonicum:* bll1796-bll1844; *B. elkanii:* GenBank accession number FM162234; *R. etli:* RHE_PD00051-RHE_PD00067; *C. taiwanensis:* RALTA_B1250-RALTA_B1267. The *tts* cluster of *S. fredii* HH103 is not presented since it is identical to the *S. fredii* USDA257.

In general, the genes encoding the T3SS core components of Rhc-I subgroup are organized, based on gene architecture, in three distinct regions, a central one (region I) and two regions (II and III), whose position in relation to the central one is variable depending on the species (Figure 3). The central region is the biggest one and contains most of the genes coding for core T3S components in the transcriptional order *nopB, rhcJ*, *nopU, rhcL*, *nopZ*, *rhcN rhcQ*, *rhcR*, *rhcS*, *rhcT*, *rhcU.* These genes possibly constitute an operon since a *tts* box is upstream of *nopB* (Perret et al., 2003; Marie et al., 2004; Wassem et al., 2008). The central region is highly conserved both in gene content and organization in all rhizobial species containing functional T3SSs. In addition, upstream of *nopB* is *rhcC1* in the opposite direction and without an obvious *tts* box in its upstream region. Despite the absence of known consensus promoter element motifs, such as *tts* boxes, upstream of some genes, this cannot rule out their TtsI- or flavonoid-dependent expression.

The regions II and III display variability both in gene content and organization and are divergently oriented in Rhc-I *tts* clusters. In *M. loti* and *S. fredii* strains, regions II and III are located downstream of *rhcC1 and rchU*, respectively, while in Bradyrhizobia are vice versa. Region II harbors the genes *ttsI*, *rhcC2* and *y4xK* coding for a putative lipoprotein (Viprey et al., 1998; Marie et al., 2004). The *ttsI* (formerly *y4xI*) is preceded by a *nod* box and encodes the transcriptional regulator of T3SS genes in rhizobia (Viprey et al., 1998; Krause et al., 2002; Marie et al., 2003, 2004; López-Baena et al., 2008; Sánchez et al., 2009). Region III contains a unit with the genes *nopC*, *nopA*, *rhcD*, *rhcV*, and *y4yS* which may constitute an operon because a *tts* box is only present upstream of *nopA* and the intergenic region between *nopA* and *rhcD* does not contain other discernible transcriptional motifs (Krause et al., 2002).

The *tts* clusters of *S. fredii* NGR234, USDA257, and HH103 have identical genetic organization, high nucleotide sequence conservation (98–99%) even in the intergenic regions, while the protein sequence similarities range from 94 to 100%. In contrast, the corresponding protein similarities in *M. loti* MAFF303099 (*M. loti*) and *B. japonicum* USDA110 (*Bja* USDA110) range between 63–95% and 41–83%, respectively (Krishnan et al., 2003; de Lyra Mdo et al., 2006). Although, *tts* clusters belonging to Rhc-I subgroup share similar gene arrangement, minor diversifications exist due to the presence or absence of some genes in regions II and III (Figure 3).

### Diversifications within Rhc-I gene clusters

In *S. fredii* strains, the region between *nopX* and *nopL* carries a curious group of genes: *y4yB*, *y4yA*, *y4xP*, *y4xO*, *y4xN*, and *y4xM*, (Perret et al., 1999; Krishnan et al., 2003; Weidner et al., 2012), which is absent in *M. loti*, *Bja* USDA 110, and *B. elkanii* (Figure 3). Although the relation of these genes with the T3SS is not yet known, several findings indicate that this gene cluster may be co-regulated with *nopX* based on the following indications: (i) absence of a *tts* box in the upstream region of *y4yB*, (ii) no transcriptional termination signals are present in the intergenic region lying between *nopX* and *y4yB*, and (iii) expression of *y4xP* is flavonoid-dependent (Perret et al., 1999; Streit et al., 2004; Lorio et al., 2006). The role of these genes remains enigmatic since mutations in three of the ORFs (*y4yA*, *y4yB*, and *y4xP*) did not affect the production and secretion of Nops (Jiang and Krishnan, 2000; Lorio et al., 2006). Additionally, *y4xP* codes for a cysteine synthase, a pivotal enzyme in sulfur assimilation and despite its location in the T3SS locus, the *y4xP* product is not a type III secreted protein. Interestingly, inactivation of *y4xP* in USDA191 enhanced the ability of the mutant to nodulate “McCall” soybean (Lorio et al., 2006). The presence of this gene cluster in the midst of T3SSs in *S. fredii* strains and in the plant pathogenic bacterium *Erwinia carotovora* suggests that they might originate from horizontal transfer from other bacteria (Streit et al., 2004). In *M. loti*, the T3E genes, *nopP* (formerly *y4yP*) and *nopL* (formerly *y4xL*) are missing. Homologs of *nopP* and *nopL* genes are also absent from phytopathogenic bacteria and are considered as rhizobial-specific (Bartsev et al., 2003, 2004; Ausmees et al., 2004).

The *tts* cluster of *Bja* USDA 110 displays more notable differences in comparison with those of *S. fredii* and *M. loti* strains. A distinct feature of the *Bja tts* cluster is the presence of several ORFs that are absent in other rhizobial species. Firstly, the region II encompasses two additional ORFs upstream of the *ttsI* (Figure 3). One is a putative T3E gene, called *nopAM* (Kimbrel et al., 2013) and the other (*bll1845)* is predicted to encode an unknown protein. The *nod* box does not reside upstream of *ttsI*, as in other rhizobial species, but upstream of *bll1845* (Krause et al., 2002). In addition, region II lacks *nopX* and no homolog is present in the rest of the *Bja* genome (Göttfert et al., 2001), while unique putative T3E genes are located in, such as *nopAR* (Yang et al., 2010b; Kimbrel et al., 2013) and *bsr1831* (Zehner et al., 2008). This region is also rich in transposases and mobile elements suggesting that may be the gene content has resulted from gene rearrangements. Similar to region II, region III contains several ORFs that might be unique in *Bja* USDA 110, while the putative operon containing the genes for *rhcD* and *rhcV* is slightly different in gene content. A *nopL*-like gene is located downstream of *rhcC1* and is predicted to encode a protein of smaller size (167 aa) in comparison to that from *S. fredii* strains (338 aa) (Krishnan et al., 2003; Ausmees et al., 2004; Rodrigues et al., 2007). An ORF annotated as *bsl1808* coding for a hypothetical protein of 67 aa is located downstream of the *nopL*-like gene. However, an ORF (annotated as *id211*) coding for a putative 91 aa polypeptide has been identified within this region (Göttfert et al., 2001). The *id211* is supposed to be the homolog of *nopC* (Deakin et al., 2005), for which no annotation has been reported so far in *Bja* USDA 110. Moreover, a *tts* box is present 83 bp upstream of *bsl1808* and overlaps with the translation start codon of the putative *id211* (Zehner et al., 2008). Future studies are needed to clarify this uncertainty. In region III, three unique genes are present. The first is the *nopE1* (*blr1806*) coding for a characterized T3E protein (Wenzel et al., 2010; Schirrmeister et al., 2011), an ORF (*bll1805*) coding for a hypothetical protein, but without an obvious *tts* box in its upstream region and *nopH* (*blr1804*) coding for a putative T3E protein (Hempel et al., 2009; Kimbrel et al., 2013). Downstream of *nopH* is located a *nopA* homolog (Krause et al., 2002; Marie et al., 2003), which is not yet annotated in the NCBI database.

### Rhc-II gene clusters

**Figure 4:**
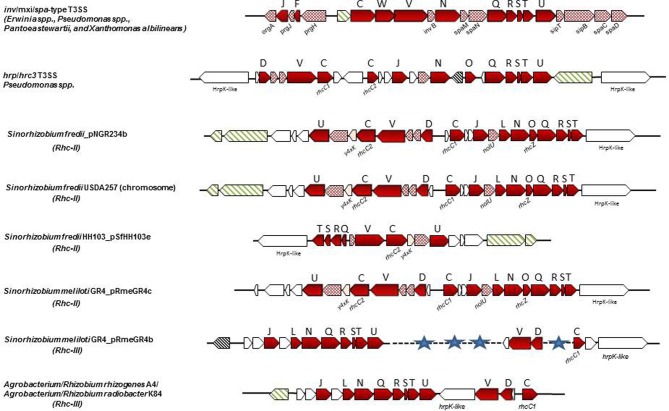
**Non-canonical T3SS gene clusters in phytopathogenic bacteria and rhizobial species**. Genes are represented according to the key of Figure 1. The locus tags for *inv*/*mxi*/*spa*-type T3SS are as follows: *E. amylovora* CFBP1430: EAMY_0771-EAMY_0792 (T3SS-II) and EAMY_1573-EAMY_1593 (T3SS-III); *Pantoea stewartii stewartii* DC283: *CKS_4544-CKS_4519; Xanthomonas albilineans* GPE PC73: XALc_1472-XALc_1511. The locus tags for *hrp*/*hrc3* in *Psp* 1448A are: PSPPH_2515-PSPPH_2540. The locus tags of each *tts* cluster are as follows: *S. fredii* NGR234a: NGR_b22750-NGR_b23010; *S. fredii* USDA257: USDA257_c21410-USDA257_c21670; *S. fredii* HH103_pSfHH103e: SFHH103_04955-SFHH103_04964; *S. meliloti* GR4_pRmeGR4c: C770_GR4pC1282-C770_GR4pC1261; *S. meliloti* GR4_pRmeGR4b: C770_GR4pB203-C770_GR4pB213, C770_GR4pB244-C770_GR4pB246, C770_GR4pB251-C770_GR4pB253; *A. radiobacter* K84: Arad_8755-Arad_8778; *A. rhizogenes* A4: arA4DRAFT_00042330-arA4DRAFT_00042490. The *tts* cluster of *R. leguminosarum bv. phaseoli* 4292 (Rleg18DRAFT_6856-Rleg18DRAFT_6875) is not presented since it is identical in gene content and organization to that of *R. etli* strains (Figure 3).

The *tts* clusters of the Rhc-II group are so far found only in Sinorhizobia (Table 2), are lacking the typical regulatory elements of Rhc-I group gene clusters and their role is yet unknown. Several Sinorhizobial species carry a functionl T3SS belonging to Rhc-I group and a second one belonging to Rhc-II group. In *S. fredii* NGR234, the T3SS located on the symbiotic plasmid pNGR234a (T3SS-I) affects nodulation in many hosts (Deakin and Broughton, 2009), while a second one (T3SS-II) resides on plasmid pNGR234b (Schmeisser et al., 2009). The latter contains 22 genes (NGR_b22800 to NGR_b23010) with twelve of them encode conserved T3SS components suggesting that it might be functional (Figure 4). However, neither *nod* nor *tts* boxes were detected in the promoter regions of these genes and the transcriptional control of the major operon of the T3SS-II cluster is weak and independent of flavonoids and/or the presence of a functional TtsI suggesting that different regulatory elements may control this T3SS locus (Bakkou and Perret, 2008; Schmeisser et al., 2009). In addition, a non-polar mutant lacking the region between *rhcC1-rhcN* (NGR_b22890-NGR_b22950) did not significantly affect the nodulation on several hosts (Bakkou and Perret, 2008; Schmeisser et al., 2009). Interestingly, a recent transcriptomic analysis (RNA-sec) of NGR234 bacteroids in morphologically distinct nodules of two legume hosts (*Vigna unguiculata* and *Leucaena leucocephala*) revealed that several T3SS-I locus genes were differentially expressed, while *nopP*, *nopX* and *nopL* were specifically upregulated in *L. eucocephala nodules.* Notably, T3SS-II locus genes were also upregulated, although the fold of induction was much higher in *V. unguiculata* bacteroids. In particular, all T3SS-II genes (NGR_b22800-NGR_b23010) or 8 out of 22 were significantly upregulated in bacteroids from indeterminate *L. leucocephala* and determinate *V. unguiculata* nodules, respectively, compared to the free-living bacteria (Li et al., 2013). It is worth noting that inactivation of T3SS-I did not affect the nodulation of *L. leucocephala* and *V. unguiculata* (Viprey et al., 1998). In light of these findings, T3SS-II seems to be functional depending either on the host or even the nodule type. Future studies are needed to shed light on the possible role of T3SS-II during nodulation in different plant hosts.

Similarly, *S. fredii* HH103 carries two T3SSs, one on the symbiotic plasmid pSfrHH103d (T3SS-I) which is functional (de Lyra Mdo et al., 2006; López-Baena et al., 2009) and a second one on plasmid pSfrHH103e (T3SS-II), which harbors genes for the production of diverse surface polysaccharides (Margaret et al., 2011). The T3SS-II is similar with a part of the second T3SS of NGR234 (Figure 4). *S. fredii* USDA 257 also carries a second T3SS which is located on the chromosome and is almost identical to the T3SS-II of NGR234 in respect to gene content and genetic organization but its role is also unidentified (Schuldes et al., 2012).

### Rhc-III gene clusters

The Rhc-III group contains the T3SSs found in the two sequenced *R. etli* strains (Table 2). The T3SS cluster is located on the symbiotic plasmid p42d of *R. etli* CFN42 and on plasmid pB of *R. etli* CIAT 652 (González et al., 2006; Gazi et al., 2012). The organization of the *rhc* genes is also different from that in other rhizobia (Figure 3). In addition, some of the core components of the T3SS that are present in other rhizobia and phytopathogenic bacteria were not detected on Rhc-III *tts* clusters. For example, the *rhcC2 is* absent in both *R. etli* strains (González et al., 2006; Gazi et al., 2012). In addition, the lack of discernible *tts* boxes in the upstream regions of *rhc* genes and the absence of homology between the TtsI and the putative transcriptional regulator (RHE_PD00067) located at the 3′- end of the cluster suggests that the T3SS of *R. etli* strains might not be regulated similarly to the T3SS of other rhizobia. Interestingly, a gene associated with the *rhc* genes is *hrpW* and it is highly conserved among diverse *R. etli* strains (Fauvart et al., 2009). Remarkably, the draft rhizobial genomes of *R. leguminosarum* bv. *phaseoli* 4292, FA23, and *R. leguminosarum* CCGE 510 harbor genes coding for core T3SS components, while no components of the T3SS have been found in the published genome sequences of *R. leguminosarum* biovars (bv. viciae 3841, bv. *trifolii* WSM2304 and bv. trifolii WSM1325). The putative T3SS genes of *R. leguminosarum* strains are located in two putative operons close to each other and are highly similar in content and gene organization with those found in *R. etli* strains. Moreover, next to the T3SS gene cluster, there are two genes coding for proteins similar to members of StcC and SctO families and a gene coding for a putative transcriptional regulator assigned to COG1396. The *R. leguminosarum* T3SS gene clusters are grouped phylogenetically with those of rhizobia belonging to Rhc-III group (Figure 2, Table 2).

T3SS gene clusters belonging to Rhc-III group are also evident in the draft genome sequences of two *Agrobacterium* strains. Although, the current taxonomic status of *Agrobacterium* spp. and *Rhizobium* spp. remains confusing, a proposal was made in 2011 by the ‘Subcommittee on the taxonomy of *Agrobacterium* and *Rhizobium*’ to reclassify the biovars 2 of *Agrobacterium* into the genus *Rhizobium* (Lindström and Young, 2011). *Agrobacterium radiobacter* K84, a biocontrol agent, and *Agrobacterium rhizogenes* A4 have been proposed to be designated as *Rhizobium rhizogenes* K84 and A4, respectively. Since these bacteria are not legume-nodulating bacteria, their reclassification into the genus *Rhizobium* is not fully supported within the scientific community. In the present article the term “rhizobia” is used in the strictest sense and includes members of the genus Rhizobium. Interestingly, the complete genome of K84 (available from GenBank and from the *Agrobacterium* genome database http://agro.vbi.vt.edu/public) and the draft genome of A4 (available from DOE Joint Genome Institute) harbor a putative T3SS gene cluster (Setubal et al., 2009; Slater et al., 2009; Figure 4, Table 2). Instead, the complete genomes of the pathogenic strains *Agrobacterium tumefaciens* C58 (biovar 1), and *Agrobacterium vitis* S4 (biovar 3) lack a T3SS. Notably, the T3SSs of K84 and A4 are most similar in gene content, organization and phylogenetic position to those belonging in Rhc-III group, while some slight differences are evident (Figures 2, 4). For example, a *hrpW* homolog is missing and a gene coding for an unknown protein of 1060 aa resides in the corresponding region. Remarkably, this putative protein displays low similarity to HrpK1 proteins from *P. syringae*. In addition, a gene coding for member of SctO family is also lacking.

### β-Rhc gene clusters

The β-Rhc subgroup is represented by *C. taiwanensis*, a β-rhizobium capable of nodulating *Mimosa* species and fixing N_2_ within the nodules (Gyaneshwar et al., 2011; Table 2). Unlike α-rhizobia and β-proteobacteria (e.g., *R. solanacearum*), *C. taiwanensis* contains a distinct T3SS with a different genetic organization, but similar to that of the human opportunist *Burkholderia cenocepacia*, suggesting a common origin for these two secretion systems (Amadou et al., 2008). In addition, none of the effector genes identified in α-rhizobia or *R. solanacearum* have been detected in *C. taiwanensis*. The T3SS genes reside on chromosome 2 and are organized possibly in four operons (Figure 3). A recent study showed that, in contrast to α–rhizobia, glutamate rather than flavonoids induced T3SS expression in *C. taiwanensis* grown in minimal media (Saad et al., 2012). Interestingly, a similar T3SS induction has been reported for *Pseudomonas aeruginosa* and *R. solanacearum* (Cunnac et al., 2004). Since these experiments were performed *in vitro*, caution should be taken before making assumptions for their regulatory networks controlling these T3SSs as the *in vivo* signals that activate them remain unknown in both rhizobia and pathogenic bacteria. Despite that *C. taiwanensis* has a functional T3SS, its inactivation did not affect nodulation of its primary host *Mimosa pudica*, but it acquired the ability of establishing chronic infections and fixing nitrogen in *Leucaena leucocephala*, a species belonging to the same Mimoseae tribe as *M. pudica* (Saad et al., 2012).

## Comparison of T3SS core proteins in phytopathogenic bacteria and rhizobia

A marked difference among the T3SS clusters of phytopathogenic and rhizobial bacteria is that the gene coding for the outer membrane secretin SctC is split in two ORFs, called *rhcC1* (formerly *nolW*) and *rhcC2*, which encode the N- and C-terminal domains of HrcC, respectively. Both parts of the split gene are separately located in the *tts* clusters. An analogous situation occurs in *B. elkanii* strains in which the secretin gene is more split into three ORFs, *rhcC1*, *rhcC2*, and *rhcC3* (Okazaki et al., 2009; de Souza et al., 2012). The split secretin gene is a distinguishing feature of rhizobial T3SS clusters known so far, except for *R. etli* strains in which the *rhcC2* is absent. Similary, the *hrcC* gene of T3SS clusters of the *P. syringae* pathovars belonging to Rhc-II family subgroup is split into two ORFs, except for *P. syringae pv. tabaci* ATCC 11528 in which the *hrcC* gene is split into three ORFs (Gazi et al., 2012). Interestingly, no *tts* box is present in the promoter region of *rhcC1* of *S. fredii* NGR234, USDA 257, HH103 and *Bja* USDA 110 although the protein is considered to be part of the transport complex of the T3SS, located in the outer membrane (Viprey et al., 1998). Previous studies have shown that *rhcC1* may be constitutively expressed in *S. fredii* NGR234 (Marie et al., 2004), *Bja* USDA 110 (Krause et al., 2002) and in *S. fredii* USDA257 (Kovács et al., 1995). These features suggest that alternative regulatory signals control the *rhcC1* expression and the protein might have an additional, still unknown, function. In a similar vain, the rhizobial SctQ proteins are single polypeptides (Viprey et al., 1998) as those in *hrp*/*hrc2* T3SSs, while in *hrp*/*hrc1* T3SSs, two split genes (*hrcQa* and *hrcQb*) encode the N-and C-terminal parts which are fused into one protein (HrcQ). Lastly, proteins belonging to less conserved T3S families, but exerting regulatory roles such as the SctP, which controls the needle-length during its assembly (Journet et al., 2003), have not yet been identified in any rhizobial species suggesting that either rhizobial-specific proteins with no sequence homology but analogous roles might exist or such a regulatory mechanism might not be required for rhizobial T3SS. In addition, members of SctO family have not been identified in rhizobia of Rhc-I subgroup, but are present in rhizobia of Rhc-II subgroup. It is likely that proteins belonging to less conserved families might have considerably diverged to fulfil the structural and functional requirements of each T3SS.

## HRP Pili and rhizobial surface appendages

T3SSs from different bacteria assemble surface appendages that are considered to serve as a channel for protein delivery into host cells (translocation). The T3SS appendages of animal pathogenic bacteria are well-characterized and morphologically resemble a needle, while phytopathogenic bacteria assemble a thinner and longer filamentous appendage, called the Hrp pilus. For a detailed comparison of these systems, the reader is referred to a recent excellent overview article (Büttner, 2012). Hrp pili have been visualized by electron microscopic studies in *P. syringae*, *R. solanacearum*, *E. amylovora*, and *Xanthomonas campestris* (Roine et al., 1997; Van Gijsegem et al., 2000; Jin et al., 2001; Weber et al., 2005). The major protein subunits (pilins) are HrpA in *P. syringae* and *E. amylovora*, HrpY in *R. solanacearum*, and HrpE in *X. campestris*. Pilins form a narrow channel with comparable dimensions in diameter [*P. syringae* (6–8 nm), *R. solanacearum* (6.6 nm), *E. amylovora* (8 nm) and *X. campestris* (8–10 nm)] and can reach more than 2 μm in length (Roine et al., 1997; Van Gijsegem et al., 2000; Jin et al., 2001; Weber et al., 2005). All are small proteins (6 to 11 kDa) and share low or no similarity among species/pathovars of different phytopathogenic bacteria. Despite their sequence variability share similar physicochemical and structural properties, increased flexibility, and polymerization modes. Genetic and phylogenetic studies have provided evidence that plant pathogenic bacteria evolved functionally and structurally similar pilins to avoid plant recognition (Lee et al., 2005; Weber et al., 2005; Guttman et al., 2006; Weber and Koebnik, 2006).

By analogy to plant pathogens, rhizobia assemble pilus-like surface appendages. So far, these pili have been visualized in three rhizobial species, *S. fredii* NGR234, USDA257, and USDA191 (Krishnan et al., 2003, 2011; Marie et al., 2003; Deakin et al., 2005; Saad et al., 2005). Similar to Hrp pili, the rhizobial pilus-like appendages are thin filaments with a diameter 6–8 nm and are formed in a flavonoid- and T3SS-dependent manner. Biochemical and electron microscopic studies have shown that three proteins were mainly associated with isolated surface appendages produced by NGR234, USDA257, and USDA191: NopA, NopB, and NopX (Krishnan et al., 2003, 2011; Marie et al., 2003; Deakin et al., 2005; Saad et al., 2005). The major subunit of rhizobial pili is the NopA protein, which is found only in T3SS-possessing rhizobial species and show no homology to pilins from plant pathogens. Common features of the pilins from phytopathogenic and rhizobial bacteria are their small size ranging between 66–113 aa and 63–71 aa, respectively, and their secondary structure is predicted to consist almost exclusively from α-helices, except for their N-termini (He et al., 2004; Deakin et al., 2005; Weber and Koebnik, 2005). In contrast to phytopathogenic bacteria the pilin proteins from different rhizobial strains are more similar to each other. The NopA protein from *S. fredii* NGR234 shares 99, 65, and 42% identity to the NopA proteins from *S. fredii* USDA257, *M. loti*, and *Bja* USDA110, respectively (Marie et al., 2003). Given the small size of rhizobial pilins, their presence in all functional T3SS-containing rhizobia, and its role in pilus formation, it would be possible that NopA might be able to polymerize and form a channel as in plant pathogens. However, future studies are needed to determine whether rhizobial pilins have similar properties with their counterparts in phytopathogenic bacteria.

NopB proteins are also present only in T3SS-containing rhizobial strains, have similar size and share high similarity especially at their C-termini. NopB from NGR234 shares 98, 63, and 43% homology to NopB from USDA257, *M. loti* and *Bja* USDA110, respectively. Moreover, NopB proteins share similarity at their conserved C-termini with FlgK proteins, known minor components of flagella in many Gram-negative bacteria (Saad et al., 2005). Genetic and electron microscopic studies have shown that NopB proteins from NGR234 and USDA257 are integral parts of rhizobial pili based on the following findings: non-polar Δ *nopB* mutants of both strains are unable to secrete any Nops in culture, do not form pili and their nodulation phenotypes resemble those exhibited by Δ *nopA* and Δ *rhcN* mutants (Viprey et al., 1998; Lorio et al., 2004; Saad et al., 2005; Kim and Krishnan, 2014). Furthermore, direct interaction of NopA and NopB was demonstrated by coimmunoprecipitation assays, while direct interaction with NopX could not be confirmed, due to its toxicity after overexpression in *E. coli.* However, it was coimmunoprecipitated with NopA and NopB from isolated surface appendages (Saad et al., 2008). A possible model have been proposed for the architecture of rhizobial pilus predicting that it is mainly composed by NopA and NopB, with the latter functioning possibly as a coupling protein within the pilus, while NopX might be part of the distal end of the pilus (Saad et al., 2008).

An interesting morphological difference between Hrp pili and those produced by NGR234 was that the latter formed a highly branched network of fibrils of variable width causing the cells to aggregate (Deakin et al., 2005). This was attributed to the presence of exopolysaccharides (EPS), known to be produced in large amounts by NGR234. Indeed, a mutant (*exoY*) unable to synthesize EPS could not produce surface appendages, albeit NopA was secreted. This finding raises questions whether EPS have a stabilizing role in rhizobial pili or reflect adaptations for rhizobia to avoid detection by the plant immune system. Recently, distinct morphological differences in T3SS-dependent pili produced by two other rhizobial species, *S. fredii* USDA257 and USDA191, were also visualized (Krishnan et al., 2011). USDA191 pili were short, rod shaped with a diameter 10–13 nm, while USDA257 produced long thin filaments with a diameter 6–8 nm similar to that of NGR234. Whether these two types of pili exhibit differential abilities to deliver effector proteins into the soybean root cells awaits investigation.

## Translocators

Translocators are T3SS proteins which are considered to form a putative pore (translocon) into the eukaryotic membrane for effector protein translocation. Although the protein composition and structure of the translocon is well characterized in animal pathogenic bacteria, it is less studied in phytopathogens. Translocon proteins from animal-pathogenic bacteria are not conserved among different species, but they generally share some common functional and structural features: (i) they are secreted proteins, (ii) they are not required for effector secretion in culture through the T3SS, but contribute to effector translocation into eukaryotic cells, (iii) knockout mutants could be functionally complemented, though partially, by translocators from the same or other phylogenetically distant species, (iv) they harbor transmembrane domains, and (v) they possess the ability to form pores in artificial lipid bilayers. Several lines of evidence suggest that a similar structure may exist in phytopathogens since they possess T3SS proteins with similar features to those mentioned above. So far, as candidate translocon components are considered to be the HrpF from *X. campestris*, PopF1 and PopF2 from *R. solanacearum* and HrpK1 from *P. syringae* and *E. amylovora* (Büttner et al., 2002; Petnicki-Ocwieja et al., 2005; Meyer et al., 2006; Büttner and He, 2009). Interestingly, the contribution of these proteins to pathogenicity differs being either essential (HrpF) or dispensable (HrpK1, PopF1 and PopF2) for effector translocation. Experimental evidence reasoned this difference on the possible additive effects of harpins or harpin-like proteins of *P. syringae* and *E. amylovora* in effector translocation (Petnicki-Ocwieja et al., 2005; Meyer et al., 2006; Kvitko et al., 2007; Bocsanczy et al., 2008). Based on genetic analyses and on putative or proven functional properties of harpins from *P. syringae*, it has been suggested that they may act together to form translocation complexes (Petnicki-Ocwieja et al., 2005; Kvitko et al., 2007). For example, several harpins are two domain proteins (HrpW of *E. amylovora*, HrpW1 and HopAK1, and PopW of *R. solanacearum*) and contain pectate lyase-like domains that suggest potential interactions with plant cell wall components (Charkowski et al., 1998; Kim and Beer, 1998; Li et al., 2010). Other harpins have been found to associate with artificial lipid bilayers and to form pores (Lee et al., 2001; Racape et al., 2005; Engelhardt et al., 2009). A similar property is also exhibited by the translocator protein HrpF of *X. campestris* pv. *vesicatoria* (Büttner et al., 2002). It is remarkable that the ability of forming pores, albeit in synthetic membranes, occurs either in putative translocators or harpins in *X. campestris* and *P. syringae*, respectively. Considering that HrpF is essential for effector translocation while HrpZ is not, it may suggest that the translocon function might be determined by the allocation of functionalities in different proteins depending on the species.

In contrast to phytopathogenic HrpW homologs (*E. amylovora* HrpW, *P. syringae* HrpW1 and HopAK1, and *R. solanacearum* PopW), the *R. etli* HrpW exhibits pectate lyase activity (Fauvart et al., 2009; Choi et al., 2013). Comparative sequence analysis showed that the *R. etli* HrpW groups together with homologs of phytopathogenic bacteria suggesting a common ancestry, albeit they differ in critical amino acids essential for the pectinolytic activity of the *R. etli* HrpW. These aminoacids are conserved between characterized pectate lyases and *R. etli* HrpW but not in phytopathogenic homologs, with the exception of *Acidovorax avenae*, which could account for the lack of pectinolytic activity of HrpW homologs from phytopathogenic bacteria (Fauvart et al., 2009). Remarkably, phytopathogenic HrpW homologs are secreted through the T3SS (Charkowski et al., 1998; Kim and Beer, 1998; Li et al., 2010), while the *R. etli* HrpW was not detected in extracellular milieu following growth of *R. etli* in minimal media (Fauvart et al., 2009). However, it is not known whether the *R. etli* T3SS genes are induced in such media which could account for this discrepancy.

The HrpW homologs in both symbiotic and pathogenic bacteria share similarities in respect to their contribution on the symbiotic and virulent phenotypes, respectively. Despite the fact that *hrpW* is induced during the early stages of root infection and inside nitrogen-fixing nodules, this induction is flavonoid-independent and it is not essential for symbiosis of *R. etli* with the common bean *Phaseolus vulgaris* (Fauvart et al., 2009). It is worth noting that *R. etli* posseses two HrpK-like proteins in the T3SS cluster which share low homology with HrpK proteins from *P. syringae* pathovars and *Erwinia* species. Thus, it is likely that the role of HrpW in symbiosis, if any, could be masked by the function of this protein. By analogy, the *P. syringae* HrpW homologs (HrpZ1, HrpW1, and HopAK1) exhibit functional redundancy and possess a minor role in virulence (Charkowski et al., 1998; Gaudriault et al., 1998; Kim and Beer, 1998; Kvitko et al., 2007; Li et al., 2010).

So far, neither *hrpW* nor *hrpK* homologs have been reported in other T3SS-containing rhizobia, although HrpK-like proteins may be encoded by the atypical T3SS gene clusters of some rhizobial species (Figure 4, author's unpublished data). However, NopX (formerly NolX) displays similarity to HrpF, but the possible role of NopX as a translocator has not yet been studied (Huguet and Bonas, 1997). Interestingly, Δ *nopX* mutants of NGR234 were able to secrete Nops in culture (Marie et al., 2003; Deakin et al., 2005). This secretion-deficient phenotype is reminiscent of that displayed by mutants in translocators from phytopathogenic bacteria. For example, secretion of *P. syringae* effectors in culture was not affected in a polymutant deleted in harpin genes and *hrpK1* (Kvitko et al., 2007). Similarly, *popF1* and *popF2* mutants of *R. solanacearum* did not impair effector secretion (Meyer et al., 2006). Although *Bja* USDA110 genome does not contain *nopX*, it possesses a gene (*blr1993*) coding for a putative polygalacturonase which is inducible by the plant isoflavonoid genistein (Caldelari Baumberger et al., 2003) and its product is secreted (Hempel et al., 2009). Recently, *blr1993* was classified as a candidate T3E gene called *nopAC*, based in the presence of a *tts* box in its upstream region and the positive phenotype in the translocation assay (Kimbrel et al., 2013). No T3E proteins have been identified so far with a putative polygalacturonase domain in T3SS-containing rhizobia. Nevertheless, the presence of cell wall-degrading enzymes in *R. etli* and *B. japonicum* suggests that they might contribute to the symbiotic process, although the link between T3SS and HrpW in *R. etli* remains to be addressed. Taken together, these similarities possibly suggest that both pathogenic and symbiotic bacteria use similar strategies in initial stages of infection, while the differences may reflect adaptations to different plant hosts.

## Atypical type III secretion systems

Many Gram-negative bacteria contain either non-canonical T3SSs with differences in gene composition, structure, regulation and function or more than one T3SSs. For example, *Salmonella* spp. possess two T3SSs which are used in different stages of infection (Hansen-Wester and Hensel, 2001). Intriguingly, *Burkholderia* spp. such as *B. pseudomallei*, harbor three T3SSs: one SPI-1-like T3SS and two Hrp-like T3SSs with the first one functioning in animal hosts and the second ones in plant hosts (Rainbow et al., 2002; Stevens et al., 2002, Figure 1). Similarly, several strains of plant pathogenic bacteria and rhizobial species carry more than one T3SSs. Most additional T3SSs are differentiated from the typical T3SSs by the lack of some structural components and/or regulatory elements, while their role in interactions with plants is yet unknown.

## Rhizobia

To date, multiple T3SSs have only been found in *Sinorhizobium* species. As described above *S. fredii* NGR234, USDA257 and HH103 possess two T3SSs, one belonging to Rhc-I subgroup (Figures 2, 3, Table 2) and a second one belonging to Rhc-II subgroup (Figures 2, 4, Table 2). Recently, comparative genomics of 48 *Sinorhizobium* strains revealed the presence of T3SS gene clusters in some strains of *S. meliloti*, *S. saheli*, *S. fredii*, and *S. terangae* (Sugawara et al., 2013). All *tts* clusters contain the main operon *rhcJ-nolUV-rhcNQRST* but the overall gene organization is different and the identified T3SS clusters are classified into three types: a, b, and c, which belong to Rhc-II, Rhc-I, and Rhc-III subgroups, respectively (Figure 2). Notably, the T3SSs are not widely distributed in *S. meliloti* strains since 9 out of 33 sequenced strains possess a T3SS (Sugawara et al., 2013, Table 2). Interestingly, none of *S. meliloti* T3SS-containing strains harbor the typical *tts* gene cluster (Rhc-I) found so far in strains of *Sinorhizobium* species, but contain a Rhc-II T3SS, which is similar to that located on plasmid pNGR234b (Table 2). From the nine *S. meliloti* strains studied by Sugawara et al. (2013), only the strain *S. meliloti* M195 carries two T3SSs belonging to Rhc-II and Rhc-III (Table 2). Furthermore, the genome of *Sinorhizobium meliloti* GR4 also contains two T3SS gene clusters, one located in the symbiotic megaplasmid pRmeGR4c and the second one in the accessory plasmid pRmeGR4b (Martínez-Abarca et al., 2013). The former belongs to the Rhc-II subgroup and the latter to the Rhc-III (Figure 2). Remarkably, the main operon (*rhcJ-nolUV-rhcNQRST*) of the T3SS located in the megaplasmid is separated from the genes coding for members of SctV, D, and C families with a long region (~30 kb) rich in mobile elements and it could be considered as atypical one in a sense (Figure 4).

*S. terangae* USDA 4894 also harbors two T3SSs belonging to Rhc-I and Rhc-III (Sugawara et al., 2013, Figure 2, Table 2). The latter resembles that of *R. etli* strains, except that a putative gene, instead of *hrpW*, is lying between *rhcU* and *rhcV* that encodes a hypothetical protein. A common feature of Rhc-II and Rhc-III T3SSs found in *S.meliloti, S. saheli*, and *S. terangae* c is the lack of known *nop* genes coding for components of T3SS-dependent surface appendages (NopA, NopB, and NopC) or effector proteins. Moreover, no *ttsI* genes were detected in any of these *tts* clusters.

Despite that the T3SS of *B. elkanii* USDA 61 contains all the core T3SS genes and the regulatory elements that are present in the typical *tts* gene clusters of Rhc-I subgroup, it exhibits a novel combination of genes with a hybrid genetic organization displaying similarities with the *tts* clusters of *B. japonicum* and NGR234 (Okazaki et al., 2009; Figure 3, Table 2). Similarly, the draft sequence of the genome of *B. elkanii* 587 revealed the presence of a *tts* gene cluster similar to that found in *B. elkanii* USDA 61 (de Souza et al., 2012). Both *B. elkanii* T3SSs possess *nopX*, which is absent in the close relative *B. japonicum*, but it is present in *S. fredii* strains (Figure 3). On the other hand, the ORFs upstream of *rhcV* are similar to those from *B. japonicum* but are absent from *S. fredii* and *M. loti* strains. Another unique feature of the *B. elkanii* T3SS is the split of the secretin gene in three ORFs: *rhcC1*, *rhc2*, and *rhcC3*. The latter shares similarity with the central part of *rhcC1*. In addition, the *tts* cluster of *B. elkanii* USDA 61 harbors genes that they have not been found in other *tts* clusters or to database entries or show similarity to non-rhizobial genes. The T3SS of *B. elkanii* USDA 61 is functional since its inactivation resulted in different symbiotic phenotypes depending on the host (Okazaki et al., 2009). However, genistein, a known inducer of the *B. japonicum tts* cluster, did not affect the secretion of Nops suggesting that the regulation of *B. elkanii* T3SS may be different from that in other rhizobia (Okazaki et al., 2009). Indeed, a recent intriguing study revealed that *B. elkanii* T3SS is capable of activating host nodulation signaling in the absence of Nod factors (NFs) and leguminous receptors (NFRs), which are considered to be key factors for symbiosis initiation (Okazaki et al., 2013). Besides, the absence of root-hairs and infection threads on the soybean roots inoculated with USDA61 suggested that *B. elkanii* T3SS may promote intercellular infection by analogy to *Salmonella* infection process. These findings show that *B. elkanii* T3SS is peculiar not only in genetic organization but also in function. It is worth mentioning that T3SSs from other rhizobial species are considered to be involved in the release of rhizobia from infection threads (Krishnan, 2002; Deakin and Broughton, 2009; Okazaki et al., 2010). Altogether these observations unveil that rhizobial T3SSs may contribute differentially in the nodulation process depending either on the rhizobial species or on the particular combination of symbiotic partners.

## *Pseudomonas* spp.

*Pseudomonas syringae* pv. *phaseolicola* 1448A *(Pph* 1448A) carries a second T3SS gene cluster, which is phylogenetically clustered together with those found in rhizobia, while individual homologs are found in *Photorhabdus*, *Aeromonas* spp. and in *P. aeruginosa* (Joardar et al., 2005; Gazi et al., 2012) This T3SS is suggested to be called *hrp*/*hrc3*, since it defines a distinct lineage in the Rhc family (Figure 1). Although it contains genes coding for all essential T3SS core components, its function is yet unknown and it is possibly unable to translocate effectors into plant cells (O'Brien et al., 2011). It neither harbors *hrp* promoter boxes in the upstream regions of T3SS genes nor genes coding for HrpL-like transcriptional regulators, albeit a gene coding for a putative transcriptional regulator is located at the 3′ end of the cluster (Figure 4). The absence of typical T3SS regulatory elements suggests that it may be controlled by alternative regulatory cirquits. The presence of this distinct T3SS has also been revealed in the draft genomes of several *P. syringae* pathovars with almost identical gene content and genetic organization to those found in *Pph* 1448A (Figure 1, Table 1), while it is absent in other pathovars, such as *P. s.* pv. *tomato* DC3000, and *P. s.* pv. *syringae* B728A (Reinhardt et al., 2009; Studholme et al., 2009; Green et al., 2010; Rodríguez-Palenzuela et al., 2010; Baltrus et al., 2011; Qi et al., 2011; O'Brien et al., 2012; Lefort et al., 2013). Future work is needed to shed light on the role, if any, and the evolutionary history of these T3SSs.

Another atypical T3SS was unveiled by genome sequencing of *P.* pv. *syringae* 642 (*Psy642*), a non-pathogenic strain isolated from a healthy leaf from an unidentified herbaceous plant (Mohr et al., 2008; Clarke et al., 2010). Although *Psy642* is closely related to *P. syringae* B728a (*PsyB728a*), it possesses a highly divergent *hrp*/*hrc* cluster which is located in a different genomic region than that occupied by the typical *hrp*/*hrc* cluster of *PsyB728a* (Clarke et al., 2010). The core components (Hrc) of this T3SS are more similar to those of *PsyB728a* than those present in other *P. syringae* strains. The distinct phylogenetic position of *Psy642* in relation to other *P. syringae* strains in the HrcU tree suggests that the *Psy642 hrp*/*hrc* cluster may have different evolutionary history and possibly it is more primitive than the canonical *P. syringae hrp*/*hrc1* clusters (Clarke et al., 2010; Figure 1).

A main structural difference of this atypical cluster is the lack of *hrpS* coding for a response regulator which together with HrpR activate expression of HrpL, the master transcription factor of the typical *hrp*/*hrc* clusters present in other *P. syringae* strains (Xiao and Hutcheson, 1994; Xiao et al., 1994). However, genes with similarity to *hrpR* and *hrpL* of *PsyB728a* are present in the same position as in the archetypal *P. syringae hrp*/*hrc* clusters. *hrp* boxes highly similar to that found in other *hrp*/*hrc* clusters (Xiao et al., 1994) are present upstream of some predicted operons but less conserved motifs were also identified in other operons of the cluster suggesting that *Psy642* HrpL might exhibit a slightly different DNA binding specificity (Clarke et al., 2010). Another remarkable difference of this new cluster is the lack of *hrpA* coding for the major subunit of Hrp pili, while an open reading frame (ORF) of unknown function has occupied the conserved genome position of *hrpA*. The *Psy642 hrp*/*hrc* cluster also lacks T3SS translocators found in other *P. syringae* strains, except for *hrpZ*, raising questions about the efficiency of effector translocation. Additionally, the *Psy642 hrp*/*hrc* cluster contains a very limited effector repertoire carrying only homologs of *avrE*, *hopM1*, and *hopB*, though very distantly related to their homologs in other *P. syringae* strains (Clarke et al., 2010; O'Brien et al., 2011). Flanking the cluster, there are two ORFs possibly organized in an operon that is preceded by a *hrp* box and code for putative effectors since they possess the physicochemical features of known T3Es. At the left border of the cluster, two ORFs are present: one carries a *hrp* box and codes for a protein similar to the effector ExoY of the opportunistic animal pathogen *P. aeruginosa* and another one, without a *hrp* box, codes for a putative lytic transglycosylase (candidate translocator), but lacking features typical of T3Es. Lastly, an ORF located in a different genomic location resembles the T3E ExoU of *P. aeruginosa*, although it does not harbor the features typical of T3Es and no *hrp* box is present. In contrast to other atypical T3SSs, the *Psy642* T3SS appear to be constitutively expressed and functional but it does not play a major role during *in planta* growth. *Psy642* was able to ectopically express the HrpL-dependent promoter of *avrPto* in rich and minimal media and to translocate AvrRpt2 into *A. thaliana* cells (Clarke et al., 2010).

Atypicall *hrp*/*hrc*-like clusters similar to that of *Psy642* appear to be common among *P. syringae* strains isolated from healthy plants and they belong to a distinct phylogenetic subgroup within group 2 of the *P. syringae* complex (Hwang et al., 2005; Mohr et al., 2008). All these strains do not induce an HR and do not cause disease on any tested plant species (Mohr et al., 2008). Despite their apparent lack of pathogenic lifestyle, the presence of genes encoding virulence factors, such as T3SS and phytotoxins, could not exclude the possibility of being pathogenic either on plant or non-plant hosts.

An unusual case, but different from those described above, is the polymorphic T3SS of *Pseudomonas viridiflava* (Araki et al., 2006). Two structurally distinct and highly divergent paralogous T3SSs are found in different chromosomal locations, but only one is present in each isolate. One has the typical tripartite organization present in the *P. syringae hrp*/*hrc* clusters, named T-PAI (tripartite pathogenicity island). The second one, named S-PAI (single pathogenicity island), is a single *hrp*/*hrc* locus similar in nucleotide sequence and gene structure to the *P. cichorii* (Hojo et al., 2008). *P. viridiflava* strains carrying any of the two paralogous T3SSs exhibit distinct differences in virulence and host specificity. Araki et al. (2006) have suggested that the maintenance of the dual PAI system may be attributed to the interaction with different hosts. The S-PAI and *P. cichorii* T3SSs lack the flanking EEL and CEL loci found in the canonical *hrp*/*hrc1* clusters, while the genes coding for AvrE1 and its chaperone AvrF1 are lying between *hrcU* and *hrcV*. Besides, both T3SSs are phylogenetically related to each other and distinct from the canonical *hrp*/*hrc1* clusters indicating a possible different evolutionary history, whereas they are closely related with those found in soft rot bacteria such as *Erwinia* spp. (Figure 1). Interestingly, the *P. cichorii* T3SS affects virulence in a host-dependent manner as being essential for the interaction with eggplant but not with lettuce. Hojo et al. (2008) suggested that *P. cichorii* likely exhibits either biotrophic or necrotrophic lifestyle depending on the host, which may account for the differential contribution of T3SS in different hosts.

Lastly, T3SSs are widespread within the *Pseudomonas fluorescens/putida* complex (Preston et al., 2001; Mazurier et al., 2004; Warmink and van Elsas, 2008; Kimbrel et al., 2010; Viollet et al., 2011). Despite their similarity in gene content and organization to T3SS gene clusters of phytopathogenic bacteria belonging in the Hrp/Hrc1 family (Figure 1, Table 1), they possess several unique features such as lack of some individual T3SS components, different regulatory mechanisms and novel roles. In a sense, these T3SSs could be considered as atypical ones in terms of regulation and function. Several studies have provided evidence for their implication in processes related with biocontrol activity, resistance to bacteriovore predation, bacterial-fungal interactions, mycorrhization and soil colonization (Preston et al., 2001; Rezzonico et al., 2004, 2005; Jackson et al., 2005; Warmink and van Elsas, 2008; Cusano et al., 2011; Mavrodi et al., 2011; Viollet et al., 2011; Maketon et al., 2012; Redondo-Nieto et al., 2013). Besides the presence of divergent *hrp*/*hrc1* gene clusters in fluorescent Pseudomonads, several strains also possess T3SSs belonging to the Rhc and Inv/Mxi/Spa families (Table 1 and references therein). For more details on these T3SSs, the reader can refer to more relevant literature, since their description is not within the scope of the present article.

## *Erwinia* spp.

Multiple and non-canonical T3SSs are also present in the genomes of *Erwinia* species. Three T3SSs have been found in the complete genomes of two *E. amylovora* strains, CFBP 1430 and ATCC49946 (Oh and Beer, 2005; Bocsanczy et al., 2008; Sebaihia et al., 2010; Smits et al., 2010b). One (T3SS-I) is similar in content and gene organization with the canonical *hrp*/*hrc* clusters of *E. amylovora* strains *Ea321* and *Ea273* (ATCC49946), known for their role in pathogenicity (Oh and Beer, 2005; Bocsanczy et al., 2008). These *hrp*/*hrc* gene clusters are also flanked by the regions HEE (Hrp effectors and elicitors), HAE (Hrp-associated enzymes), and IT (Island transfer) (Oh and Beer, 2005). The other two T3SSs (T3SS-II and T3SS-III; Figure 1) are related to the *inv*/*mxi*/*spa* gene clusters of animal pathogens (e.g., *Salmonella* and *Shigella* spp.) and also resemble to the additional T3SSs found in the phytopathogens, *E. pyrifoliae* DSM 12163 (Smits et al., 2010a), *E. amylovora* 273 (Bocsanczy et al., 2008), *E. amylovora* 321, in the epiphytic strain *E. tasmaniensis* Et1/99 (Kube et al., 2010), in the insect endosymbiont *Sodalis glossidinius* (Dale et al., 2001) and in bacteria of the *P. fluorescens* group, such as *P. fluorescens* Q2-87 (Loper et al., 2012), *P. fluorescens* F113 (Redondo-Nieto et al., 2013), *P. fluorescens* HK44 (Chauhan et al., 2011), while differences in individual T3SS components have been reported (Barret et al., 2013). Furthermore, incomplete T3SSs of the Inv/Mxi/SpaS type have been found in several strains of *Pseudomonas* spp. which interact with non-plant hosts such as mushrooms, nematodes or are soil colonizers with yet unknown biotic relationships (Barret et al., 2013, Table 1). Although the role of these T3SSs is still unknown, their presence in non-pathogens may be indicative of their contributions to biotic associations with non-plant hosts or to the environmental fitness of bacteria. Indeed, deletion of both T3SSs (II and III) from the *E. amylovora* genome demonstrated that they are not essential for virulence in plants (Zhao et al., 2009). On the other hand, the similarity of these T3SSs to that of the insect endosymbiont *Sodalis glossidinius* str. *morsitans* has led to the assumption that they may facilitate to as yet unknown interactions with insects. Notably, the second T3SS cluster of *Pantoea stewartii* subsp. *stewartii* belonging to the Inv/Mxi/Spa type T3SSs is required for bacterial persistence in its insect vector and subsequent transmission by the vector to host plants, while its Hrp/Hrc1type T3SS is involved in plant colonization (Correa et al., 2012; Figures 1, 4). Recent studies provide evidence that the Inv/Mxi/Spa type T3SSs found in some plant beneficial bacteria may be involved in interactions with non-plant hosts. For example, the second T3SS cluster of *P. fluorescens* F113 is induced during F113-amoeba interactions and seems to be involved in resistance to bacteriovorous protists, while the role of its Hrp/Hrc1-type T3SS is yet unknown (Barret et al., 2013). Lastly, the single T3SS of *Xanthomonas albilineans* also belongs to the Inv/Mxi/Spa type T3SSs (Marguerettaz et al., 2011; Figures 1, 4). Although its role is yet unknown, functional analysis provided evidence that it is not required for virulence in plants.

Common to all atypical T3SSs, except those of *P. viridiflava* and *B. elkanii*, is the lack of genes coding for the well-characterized transcriptional regulators, structural components of pili, effector proteins and have no apparent role in plant-microbe interactions. The similar features of these atypical T3SSs in bacteria with different lifestyles raise questions about their evolutionary origin and biological role. It is likely that they might be controlled by different regulatory networks than those controlling the T3SSs with a demonstrated role in plant-microbe interactions. These unusual T3SSs might secrete and/or translocate novel effectors exhibiting different features than those described for the well-characterized T3Es found in typical T3SSs. By analogy, surface appendages composed of different proteins might be made by these T3SSs. Remarkably, the so far characterized T3SS pili of both phytopathogenic bacteria and rhizobia consist of proteins with low or no homology to each other. Alternatively, these T3SSs may not form extracellular appendages (“needle-less” T3SS) similar to pili but they may be used only for extracellular secretion instead of effector translocation into other cells. Future studies are needed to shed light on the above issues and on the evolutionary mechanisms that have driven the acquisition and maintenance of these T3SSs in both pathogenic and symbiotic bacteria as well as to determine their role during their life cycles.

## Concluding remarks

The availability of an increasing number of genomes from T3SS-containing bacteria have unveiled an incredible diversity and complexity of T3SSs thus providing the opportunity and the challenge to better understand how this sophisticated secretion mechanism has evolved and adapted to fulfil the specific requirements of diverse bacterial species. The newly discovered systems provide the framework to define the similarities and differences of T3SSs that shape diverse biotic interactions. To date, most type III secretion research has focused on pathogenic bacteria, while few efforts have been made in symbiotic bacteria. Although rhizobial T3SSs contain most of the core type III components, the diversifications observed in members of some T3S protein families suggest that the structure and regulation of rhizobial secretion machineries have possibly adopted specific adaptations to serve the association of rhizobia with legume roots. Uncovering the specific structural and secretion regulatory components in various types of T3SSs will increase our knowledge about the mechanisms underlying the structure and function of T3SSs across phylogenetically diverse bacteria. An interesting avenue for further research will be the delineation of the T3S regulons in both phytopathogenic bacteria and rhizobia. The absence of well-known regulatory elements from various types of T3SS gene clusters denotes that transcriptional factors and *cis*-elements different from the so far known may be involved in controlling gene expression. On the other hand the presence of conserved *cis*-elements upstream of genes with no apparent relation with the T3SS implies that these genes may be co-regulated. Next generation transcriptomics promises to provide new insights into the well-known transcriptional networks that govern type III secretion.

Furthermore, the presence of novel non-canonical T3SSs with yet unknown roles in both pathogenic and symbiotic bacteria opens new avenues in T3SS research hoping to reveal fascinating strategies exploited by the bacteria to interact with their biotic environment. Study of these T3SSs may help uncover new facets of their role in the interactions with plant and non-plant hosts. Future research efforts should be directed to defining the signaling circuits that control the expression of the atypical T3SSs. Deciphering the regulatory networks will be key to the future development of novel functional assays for screening and characterizing novel effectors.

Finally, a comprehensive understanding of T3SS structure and function in diverse bacteria is expected to illuminate various aspects of pathogenesis and symbiosis related with the evolution of T3SSs in bacteria with different lifestyles and the molecular mechanisms underlying plant-microbe interactions and beyond. Moreover, this knowledge will contribute in the development of various biotechnological applications involving the design of efficient strategies to control plant diseases, the exploitation of T3SS as scaffold for protein delivery into eukaryotic cells, the improvement of the symbiotic properties of rhizobial species or even the expansion of the symbiotic potential toward non-leguminous plants of agriculture importance.

### Conflict of interest statement

The author declares that the research was conducted in the absence of any commercial or financial relationships that could be construed as a potential conflict of interest.
